# Development and temporal validation of a screening-based prediction model for suicidal ideation at first contact with university counseling services

**DOI:** 10.1186/s40359-026-05207-9

**Published:** 2026-07-25

**Authors:** Yuko Yamada, Masao Ishitsuka, Tomohisa Ohmachi, Kiun Kato, Togo Aoyama

**Affiliations:** 1 Health Care Center, Kitasato University, 1-15-1 Kitazato, Minami-Ku, Sagamihara, Kanagawa 252-0373 Japan; 2 Faculty of Education, Soka University, 1-236 Tangi-Machi, Hachioji, Tokyo 192-8577 Japan

**Keywords:** Suicidal ideation, University students, Screening, Risk prediction model, Self-esteem, Psychological distress, Temporal validation, Decision curve analysis, Counseling services, Japan

## Abstract

**Background:**

Suicidal ideation among university students is a major public health concern. Yet counseling services still lack practical tools that can identify students at elevated risk during their first clinical contact. This study aimed to develop and temporally validate a screening‑based prediction model for suicidal ideation at the time of initial presentation to university counseling services.

**Methods:**

This retrospective observational study analyzed routinely collected data from a Japanese university counseling services. Predictor variables were exclusively derived from annual mental health screening data collected before participants used counseling services. The variables included psychological distress, self‑esteem, lifestyle‑related factors, and demographic characteristics. The outcome was suicidal ideation assessed during the first counseling intake interview. We developed a multivariable logistic regression model using a development cohort of students who accessed counseling services between 2017 and 2019 and temporally validated the model in an independent cohort from 2021 to 2023. We evaluated the model performance using discrimination, measured by the area under the receiver operating characteristic curve (AUC), calibration plots, and decision curve analysis (DCA). A reference value threshold was identified using a Youden Index–based cutoff.

**Results:**

The development and validation cohorts included 1,136 and 1,286 students, respectively. The prevalence of suicidal ideation at intake was comparable across cohorts (14.0% and 14.7%). The model achieved moderate discrimination in the development cohort (AUC = 0.71) and good discrimination in the validation cohort (AUC = 0.74), with acceptable calibration in both cohorts. Higher levels of screening‑based psychological distress, particularly feelings of worthlessness, female sex, and lower self-esteem increased the odds of suicidal ideation at intake. Conversely, call-based or early outreach referral routes were associated with lower odds. DCA showed that the model provided greater net benefit than strategies that intervened in all or no students across clinically plausible threshold probabilities, including the Youden Index‑based cutoff (approximately 0.13).

**Conclusions:**

Routinely collected mental health screening data can predict suicidal ideation at the time of initial counseling contact with reasonable accuracy and potential clinical utility. Screening‑based prediction models may strengthen existing student support systems by enabling earlier identification of elevated risk and guiding proportionate intervention strategies.

## Background

Suicidal ideation: from passive desires for death to active thoughts of self-harm—is widely recognized as a critical precursor to suicide attempts and completed suicide [1–3]. Worldwide, suicide is a major public health crisis and claims more than 720,000 lives annually representing approximately 1 out of 100 deaths. For people aged 15–29 years, suicide ranks third as the leading cause of death [4]. In Japan, suicide has been the leading cause of death among youth aged 15–29 years since the late 1980s. While the overall suicide rate has declined after peaking in 2003, this demographic has demonstrated a concerning upward trend since 2016 [5]. Japan is the only G7 nation where suicide is the leading cause of death among young adults—underscoring a critical public health issue [6, 7].

Among Japanese university students, many report substantial psychological distress and reduced well-being. For example, 37.5% report life as difficult, with feelings of inadequacy and emptiness associated with suicidal ideation [8]. While Western models view emptiness as a symptom of depression or borderline personality disorder [9, 10], Japanese scholars emphasize an existential emptiness characterized by perceived insignificance and loss of *ikigai*—serving as predictors of suicidality independent of clinical diagnoses [11, 12]. The increase observed in 2016–2019 is mainly attributed to the negative impacts of social networking services, including cyberbullying and social comparison, alongside chronic school-related stressors and the absence of adequate support systems [5, 13, 14]. Recent large-scale stressors have underscored these vulnerabilities. During COVID-19, suicide rates increased among undergraduate students in Japanese national universities [15]. These examples demonstrate how external stressors exacerbate psychological distress and reveal structural weaknesses in university mental health systems.

These environmental and psychosocial pressures manifest as persistent suicidal ideation [5, 14]. As per global estimates, each suicide is accompanied by more than 20 attempts, with a high prevalence of suicidal ideation [16, 17]. International studies report prevalence rates ranging from 10 to 20% among university students [18–21]. Recent surveys in Japan indicate that approximately one out of four young adults has seriously considered suicide at certain points in life [22, 23]. This ideation functions as a critical precursor to suicidal behavior [1–3, 16], but many individuals remain isolated without seeking professional help [16, 22]. These findings highlight the need for timely identification of students at elevated risk. Early risk stratification remains a critical component of effective suicide prevention in university settings.

A substantial body of research has examined the risk and protective factors associated with suicidal ideation. Psychological distress consistently emerges as a strong predictor of suicidal ideation, whereas self-esteem functions as a robust protective factor across cultural contexts [24–29]. Several dimensions of psychological distress including worthlessness, hopelessness and depressive mood have been associated with suicidal ideation in previous studies [29–32]. However, their independent effects appear to vary after adjustment for overlapping psychological constructs and self-evaluative processes. For example, previous studies have suggested that hopelessness mediates the relationship between low self-esteem, heightened social anxiety, and suicidal ideation among university students [32]. In contrast, evidence indicates that suicidal ideation is more strongly linked to academic stress than to self-esteem in undergraduate populations [33]. Suicidal ideation has also been associated with lifestyle-related factors, including poor sleep duration (both short and prolonged sleep), physical inactivity, and unhealthy dietary patterns [34]. Longitudinal studies indicate that modifiable psychosocial and lifestyle factors observed at university admission often persist across subsequent academic years and increase the vulnerability to mental health problems [35]. These findings underscore the importance of such factors as contextual markers for early risk stratification rather than as isolated causal determinants.

Collectively, this evidence underscores the multifactorial nature of suicidal ideation, emerging from the interaction among psychological distress, reduced protective factors, lifestyle disruptions, and barriers to access to support services [36–40]. In addition, a consistent phenomenon observed across cultures is the *gender paradox of suicide*, characterized by higher rates of suicidal ideation and nonlethal attempts among women and significantly higher rates of completed suicide among men [41, 42]. Recent evidence reports that suicide rates among women have rapidly increased, and gender-specific risk factors have been increasingly recognized, underscoring the need for tailored, gender-sensitive prevention strategies [43, 44]. Despite these insights, Japanese studies examining the combined influence of these factors within an integrated predictive framework—particularly in university settings—remain scarce. This gap is especially consequential given constrained mental health resources in many institutions and the need to prioritize support for students at greatest risk.

To facilitate early identification of high-risk students, universities worldwide have implemented systematic mental health screening initiatives [45, 46]. In Western countries, programs such as the Interactive Screening Program in the United States, MHC-based screening initiatives in Canada, and StudiCare in Germany have shown efficacy in connecting distressed students to mental health services [47–49]. Similarly, Japanese universities have increasingly adopted digital health check-ups and screening instruments such as the Kessler Psychological Distress Scale (K10) to identify students with severe psychological distress [46, 50]. The K10 comprises 10 items for assessing the frequency of nonspecific psychological distress symptoms within the past month. Respondents rate the frequency of experiencing feelings such as fatigue without reason, nervousness, hopelessness, restlessness, depression, effortfulness, and worthlessness [51]. Items are rated using a five-point scale ranging from 0 (none of the time) to 4 (all of the time), with high scores indicating greater psychological distress. Compared with available tools, K10 demonstrated strong psychometric properties, brevity, and feasibility for large-scale screening, including clinical utility in identifying individuals with psychological distress [52, 53]. The K10 was selected over disorder-specific measures such as the Patient Health Questionnaire-9, because it captures nonspecific psychological distress relevant to early risk detection in nonclinical populations [54, 55]. Recent studies proposed that high scores on three subitems of the K10 (4: hopelessness; 7: depression; 10: worthlessness) predicted high levels of suicidal ideation [32, 56]. However, screening alone cannot address the practical challenge faced by university counseling services: how to prioritize limited mental health resources effectively. This challenge is particularly acute during initial counseling intake, when clinicians require brief, interpretable, and clinically actionable indicators to support rapid risk stratification.

In this context, predictive modeling offers a complementary framework for estimating the individual-level risk of suicidal ideation using information available before or at the time of first contact with counseling services. Several prediction models of suicide risk among university students have reported high discriminatory performance using machine learning and advanced statistical methods [57–60]. However, many of these models prioritize algorithmic complexity over clinical interpretability. They also rarely assess performance stability across different periods, which limits their practicality in real-world university counseling settings.

Accordingly, this study aims to develop and temporally validate a multivariable prediction model for suicidal ideation among Japanese university students accessing counseling services for the first time. The model uses routinely collected mental health screening data to predict suicidal ideation at initial counseling intake. Unlike many existing approaches, this study prioritizes interpretability, feasibility, and temporal robustness over causal inference or algorithmic sophistication alone. The study may support earlier risk stratification and more efficient allocation of mental health resources within higher education settings.

## Methods

### Study design and setting

This retrospective observational study analyzed data collected from a university counseling service in Japan. The primary aim of this study was to develop and temporally validate a multivariable prediction model for suicidal ideation at first contact with the counseling service. The study followed the TRIPOD (Transparent Reporting of a multivariable prediction model for Individual Prognosis or Diagnosis) guidelines.

### Study procedures and data sources

At the beginning of each academic year, the university administered a mental health screening survey to all enrolled undergraduate students as part of routine student support activities. The survey operated independently of counseling service use and collected data on psychological distress, self‑esteem, lifestyle factors, and demographic characteristics.

The present study focused exclusively on students who subsequently accessed the university counseling service for the first time. During routine intake procedures, students completed an intake questionnaire that assessed current suicidal ideation.

Predictor variables were derived from the annual campus‑wide screening survey. The outcome variable, suicidal ideation at initial counseling intake assessed exclusively during counseling intake and was not obtained from the screening survey.

### Participants

Participants were limited to students who accessed the university counseling service for the first time during the study periods and completed the intake questionnaire, which included an item assessing suicidal ideation, at initial contact. Two temporally distinct cohorts were defined: a development cohort (2017–2019) and a temporal validation cohort (2021–2023). Owing to disruptions related to the COVID-19 pandemic, data from 2020 were excluded.

### Demographic characteristics

The participants in the development cohort (2017–2019; *n* = 1,136) were predominantly aged 18–21 years (38.8%, 22.9%, 15.4%, and 11.5%, respectively), with women and men representing 53.6% and 46.4%, respectively. More than half of the participants (54.0%) were first-year students, while 17.8%, 13.6%, 12.8%, and 1.9% were second-, third-, fourth-, and fifth and sixth-year students, respectively. Similarly, the participants in the temporal validation cohort (2021–2023; *n* = 1,286) were predominantly aged 18–21 years (40.2%, 21.9%, 16.3%, and 12.7%, respectively), with women and men accounting for 56.2% and 43.8%, respectively. More than half (55.1%) were first-year students, followed by 17.7%, 13.8%, 11.8%, and 1.6% in the second-, third-, fourth-, and fifth and sixth-year students, respectively.

### Outcome variable

Suicidal ideation at the time of initial counseling intake was defined as the dependent variable. Suicidal ideation was assessed using a single dichotomous item included in the intake questionnaire and coded as present or absent [61]. This measure reflected current suicidal ideation at intake rather than the individual’s lifetime history.

### Predictor variables

Predictors were prespecified based on prior literature and clinical relevance and were defined and identically coded in the development and validation cohorts. All predictors were treated as risk markers rather than causal or intervention variables.

Psychological distress was assessed using three subitems of the Kessler Psychological Distress Scale (K10): worthlessness, hopelessness, and depressive mood. Each item was originally measured on a five‑point Likert scale (0–4). For the purpose of prediction modeling, responses were dichotomized a priori, with scores of 0–2 coded as low (0) and 3–4 coded as high (1).

Self‑esteem was assessed using the total score of the Rosenberg Self‑Esteem Scale (SE_total) and treated as a continuous variable [62].

Lifestyle‑related variables included sleep duration (short or long sleep duration [≤ 5 h or ≥ 8 h] versus typical sleep duration [6–7 h]), lack of regular exercise, skipping breakfast, and loss of appetite, all of which were coded as dichotomous variables. Sex was coded as female or male. The access route to the counseling service was coded as call‑based or early outreach contact versus voluntary self‑initiated access. Call‑based or early outreach contact referred to cases in which students were proactively contacted by the counseling service when routine screening identified elevated psychological distress, defined as a total score of 15 or higher on the Kessler Psychological Distress Scale (K10; range = 0–40) [63]. The analysis treats this variable as an indicator of route of access to services rather than as an intervention variable.

Additionally, multicollinearity among predictors was assessed using variance inflation factor (VIF) in the development cohort. All VIF values were below 2 and close to 1, indicating no evidence of problematic multicollinearity.

### Model development

A multivariable logistic regression model was developed using data from the 2017–2019 development cohort. All prespecified predictors were entered simultaneously into the model without variable selection or penalization (forced‑entry method). Regression coefficients were estimated using maximum likelihood estimation.

Model discrimination in the development cohort was assessed using the area under the receiver operating characteristic curve (AUC). Apparent calibration in the development cohort was evaluated using decile‑based calibration plots comparing predicted probabilities with observed outcome frequencies.

### Determination of optimal risk threshold

To identify a representative probability threshold for classifying elevated risk of suicidal ideation, the Youden Index (J = sensitivity + specificity − 1) was calculated using the development cohort. The optimal cutoff value was defined as the predicted probability that maximized the Youden Index.

Additionally, sensitivity and specificity across the range of predicted probabilities were graphically examined, and the intersection of sensitivity and specificity curves was used to visually confirm the Youden‑based cutoff.

The identified cutoff was treated as a reference value for evaluating model performance and clinical utility and was not intended to define a fixed clinical decision rule.

### Temporal external validation

Temporal external validation was performed using an independent cohort of students who accessed the counseling service between 2021 and 2023. Regression coefficients and intercepts estimated in the development cohort were applied unchanged to the validation cohort to generate predicted probabilities of suicidal ideation.

Model discrimination in the validation cohort was evaluated using the AUC. Calibration in the validation cohort was assessed using decile‑based calibration plots comparing the predicted probabilities with observed outcome frequencies. No model refitting or recalibration was performed during validation.

### Decision curve analysis

To evaluate the potential clinical utility of the prediction model in the validation cohort, decision curve analysis (DCA) was conducted. Net benefit was calculated across a range of threshold probabilities and compared with default strategies of intervening either in all students or in none.

The Youden‑based cutoff probability was superimposed on the decision curve as a reference point to examine whether the use of the prediction model conferred greater net benefit than alternative strategies at clinically relevant risk thresholds.

### Statistical analysis

Descriptive statistics were used to summarize participant characteristics. Continuous variables were summarized using means and standard deviations, and categorical variables were summarized using frequencies and proportions. Logistic regression analyses and post‑estimation procedures were conducted using Stata (ver.19.0). Missing data were minimal (< 2% across variables) and were handled using multiple imputation. Statistical significance testing was considered secondary because the primary objectives were prediction, temporal validation, and evaluation of potential clinical utility rather than hypothesis testing.

Additionally, to characterize temporal patterns in the broader screening population and in service utilization, supplementary chi-square analyses were conducted. In particular, we examined differences across academic years in (1) the proportion of students classified as high risk based on screening data and (2) access routes to counseling services (call-based/early outreach vs. voluntary). The effect sizes were quantified using Cramer’s V, and adjusted residuals were examined to identify the specific cells that contributed to the overall associations. These analyses were conducted to provide contextual interpretation of temporal changes in help-seeking behavior, supplementing the primary prediction modeling analysis.

### Ethical approval

This study analyzed anonymized data collected as part of routine university counseling service procedures. Kitasato University School of Medicine and Hospital Ethics Committee approved the study (Approval No. B23-166). All procedures adhered to the Declaration of Helsinki (2013 revision). Informed consent was obtained for the use of annual screening data, and intake questionnaire data were handled under an opt-out procedure in accordance with national ethical guidelines.

### AI-assisted language editing

A large language model (Microsoft Copilot) was used solely for English-language editing and to improve the clarity and readability of the manuscript. All AI-assisted suggestions were reviewed, verified, and revised by the authors, and they retain full responsibility for the final content. No AI tools were used for data analysis, statistical procedures, or interpretation.

## Results

### Study population

The development cohort included students who first accessed the university counseling service between 2017 and 2019, while the temporal external validation cohort included those who accessed the service between 2021 and 2023. Missing data were handled using multiple imputation; therefore, all participants were retained in the analyses.

Although the baseline characteristics of the two cohorts were broadly comparable, the proportion of students accessing counseling via call‑based or early outreach routes was higher in the validation cohort. The prevalence of suicidal ideation at intake was similar between the cohorts (development cohort, 14.0%; validation cohort, 14.7%). Detailed cohort characteristics are presented in Table 1.

**Table 1:** Baseline characteristics of students accessing university counseling services for the first time

Variables	Development cohort (2017–2019), *n* = 1,136	Validation cohort (2021–2023), *n* = 1,286
Suicidal ideation, [Outcome variable], %	14.0	14.7
Self-esteem score (SE_total), mean (SD)	22.46 (4.44)	22.80 (4.75)
Call-based access (vs. voluntary), %	64.7	75.7
Female, %	53.6	56.2
K10 worthlessness (high^*^), %	22.6	23.3
K10 hopelessness (high^*^), %	14.3	15.5
K10 depression (high^*^), %	31.0	34.3
Short or long sleep duration, %	37.9	32.1
Lack of regular exercise, %	42.9	37.1
Skipping breakfast, %	20.1	23.1
Loss of appetite, %	29.6	32.8

Baseline characteristics of the development cohort (2017–2019) and the temporal validation cohort (2021–2023) are shown. Predictor variables were obtained from a routinely conducted campus‑wide mental health screening administered prior to counseling service use, including psychological distress indicators, self‑esteem, lifestyle‑related factors, and demographic characteristics. Suicidal ideation was assessed at the time of initial counseling intake. Values are percentages unless otherwise indicated. Self‑esteem is presented as the mean (standard deviation). Kessler Psychological Distress Scale (K10) subitems (worthlessness, hopelessness, depressive mood) were dichotomized as low (scores 0–2) versus high (scores 3–4)

Additional information concerning the screening population and the flow of students to counseling service utilization is presented in Table 2. The proportion of students identified as high risk based on screening criteria remained relatively stable across academic years, ranging from approximately 10% to 13%. In contrast, patterns of service utilization varied across years. The proportion of students who attended counseling through call-based or early outreach was higher in the validation cohort (2021–2023), whereas voluntary visits were relatively more common in the development cohort (2017–2019). Supplementary chi-square analyses indicated a significant association between academic year and access route (call-based vs. voluntary) (χ^2^(5) = 55.18, *p* < 0.001), with a moderate effect size (Cramer’s V = 0.151). Adjusted residuals showed that voluntary visits were significantly more frequent in 2017–2019, whereas call-based access was significantly more frequent in 2021. Notably, although the association between academic year and screening-based high-risk classification was statistically significant, the effect size was negligible (χ^2^(5) = 32.997, *p* < 0.001, Cramer’s V = 0.035), indicating minimal variation in the underlying distribution of psychological risk across years.

**Table 2:** Annual screening population and flow from screening to counseling service utilization

Academic year and subtotal of each cohort	Target populations	Number of valid responses	Number of outreach targets (highrisk;%)	Number of high-risk interviews	Number of unresponsive students	Number of voluntary visitors
2017	5,238	4,677	577 (12.3)	250	327	142
2018	5,248	4,794	527(11.0)	252	275	135
2019	5,274	4,821	480(10.0)	233	247	124
Development Cohort	15,760	14,292	1,584(11.1)	735	849	401
2021	5,275	3,972	526(13.2)	357	169	74
2022	5,317	4,594	576(12.5)	345	231	115
2023	5,405	4,525	553(12.2)	272	281	123
Validation Cohort	15,997	13,091	1,655(12.6)	974	681	312

This table summarizes the flow of students from campus-wide mental health screening to counseling service utilization for each academic year. The total number of students targeted for screening, number of valid responses, number of students identified as high risk based on screening criteria, and subsequent engagement with counseling services (call-based outreach interviews, voluntary visits, and nonresponse after outreach) are presented. The table provides contextual information concerning the relationship between the screened population and the analytic sample used in the prediction model. In particular, it illustrates the proportion of high-risk students who did not attend counseling services despite outreach efforts, highlighting potential selection bias and temporal changes in help-seeking behavior across academic years

### Model development and performance in the development cohort

**Fig. 1:**
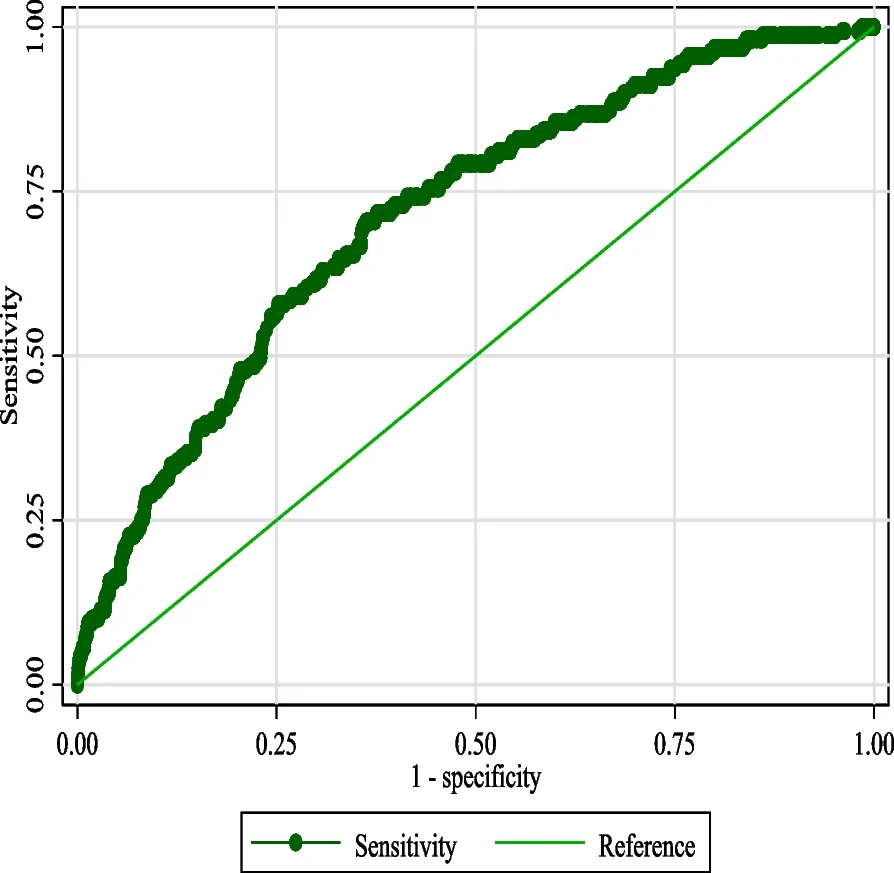
Receiver Operating Characteristic (ROC) Curve for the Prediction Model in the Development Cohort (2017–2019). The model demonstrated moderate discriminatory performance for suicidal ideation assessed at initial counseling intake, with an area under the ROC curve (AUC) of 0.71

A multivariable logistic regression model predicting suicidal ideation at initial counseling intake was developed using the 2017–2019 cohort, with all prespecified predictors entered simultaneously. In the development cohort, the model demonstrated moderate discriminatory performance, with an area under the receiver operating characteristic curve (AUC) of 0.71 (Fig. 1).

Higher levels of screening‑based psychological distress—particularly high levels of worthlessness—as well as female sex were associated with increased odds of suicidal ideation. In contrast, higher self‑esteem scores and access to the counseling service via call‑based or early outreach contact were associated with lower odds of suicidal ideation. Lifestyle‑related variables, including lack of regular exercise and skipping breakfast, were not independently associated with suicidal ideation but were retained in the model as covariates owing to their theoretical relevance and potential clinical utility.

Odds ratios, corresponding 95% confidence intervals, and *p*-values for all predictors included in the development model are shown in Table 3.

**Table 3:** Screening‑based multivariable logistic regression model predicting suicidal ideation at initial intake (Development cohort, 2017–2019)

Predictors	Odds ratio (95% CI)	*p*-value
Self-esteem (SE_total) (per 1-point increase)	0.94 (0.90–0.99)	0.014
K10 worthlessness (1)	2.04 (1.27–3.27)	0.003
K10 hopelessness (1)	1.39 (0.83–2.31)	0.207
K10 depression (1)	1.46 (0.93–2.32)	0.103
Female (1)	1.60 (1.10–2.31)	0.014
Call-based/early outreach access (1)	0.35 (0.23–0.52)	< 0.001
Short or long sleep duration (1)	1.17 (0.81–1.67)	0.404
Lack of regular exercise (1)	1.12 (0.72–1.65)	0.541
Skipping breakfast (1)	1.24 (0.80–1.93)	0.328
Loss of appetite (1)	1.32 (0.76–2.29)	0.329

Multivariable logistic regression results from the development cohort. The outcome variable was suicidal ideation assessed at the time of initial counseling intake (yes/no). All predictors were obtained from pre‑intake mental health screening. K10 subitems were dichotomized as low (scores 0–2) versus high (scores 3–4). Odds ratios (ORs) greater than 1 indicate higher odds of suicidal ideation, whereas ORs less than 1 indicate lower odds

### Determination of optimal risk threshold

Using the development cohort, a representative probability threshold for identifying individuals at elevated risk of suicidal ideation was explored using Youden’s J statistic. The Youden Index was maximized at a predicted probability of approximately 0.13, corresponding to a sensitivity of approximately 0.70 and a specificity of approximately 0.64.

This cutoff was treated as a reference value to illustrate the balance between sensitivity and specificity and was not intended to define a fixed clinical decision rule.

### Temporal external validation

Temporal external validation was conducted using the independent cohort of students who first accessed the counseling service between 2021 and 2023. Regression coefficients and intercepts estimated in the development cohort were applied unchanged to the validation cohort to generate predicted probabilities of suicidal ideation.

**Fig. 2:**
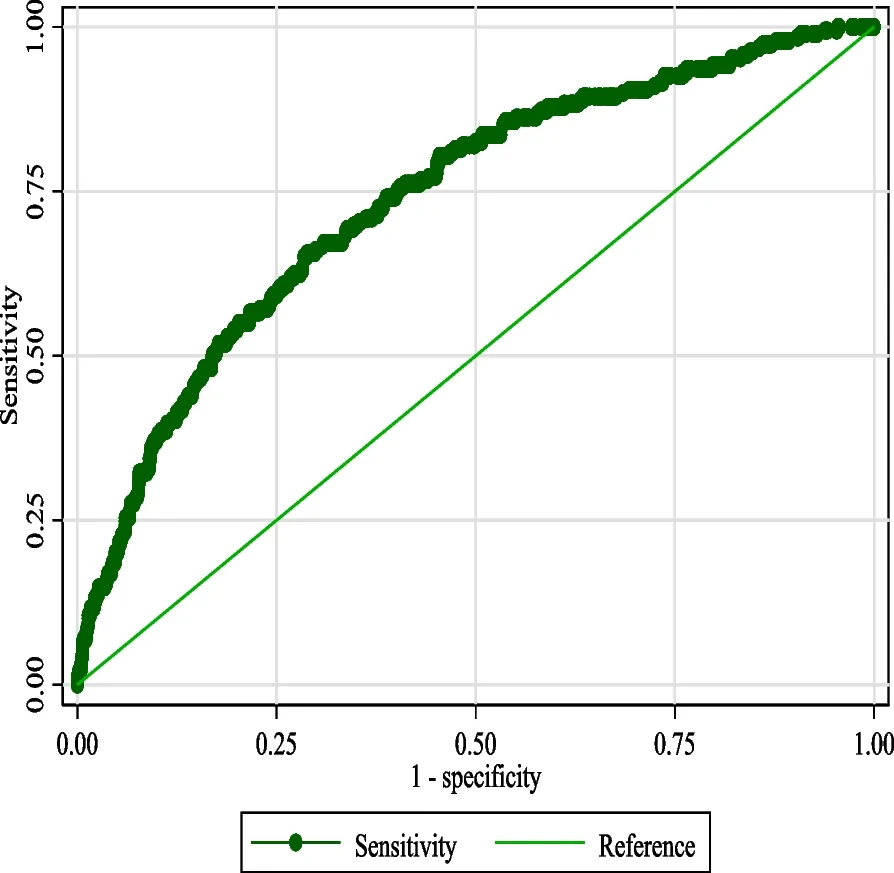
ROC Curve for the Prediction Model in the Temporal Validation Cohort (2021–2023). The model showed good discrimination in the validation cohort (AUC = 0.74), indicating that predictive performance was maintained across temporally distinct cohorts

In the validation cohort, the model demonstrated good discriminatory performance, yielding an AUC of 0.74, indicating that predictive performance was maintained across temporally distinct cohorts (Fig. 2).

**Fig. 3:**
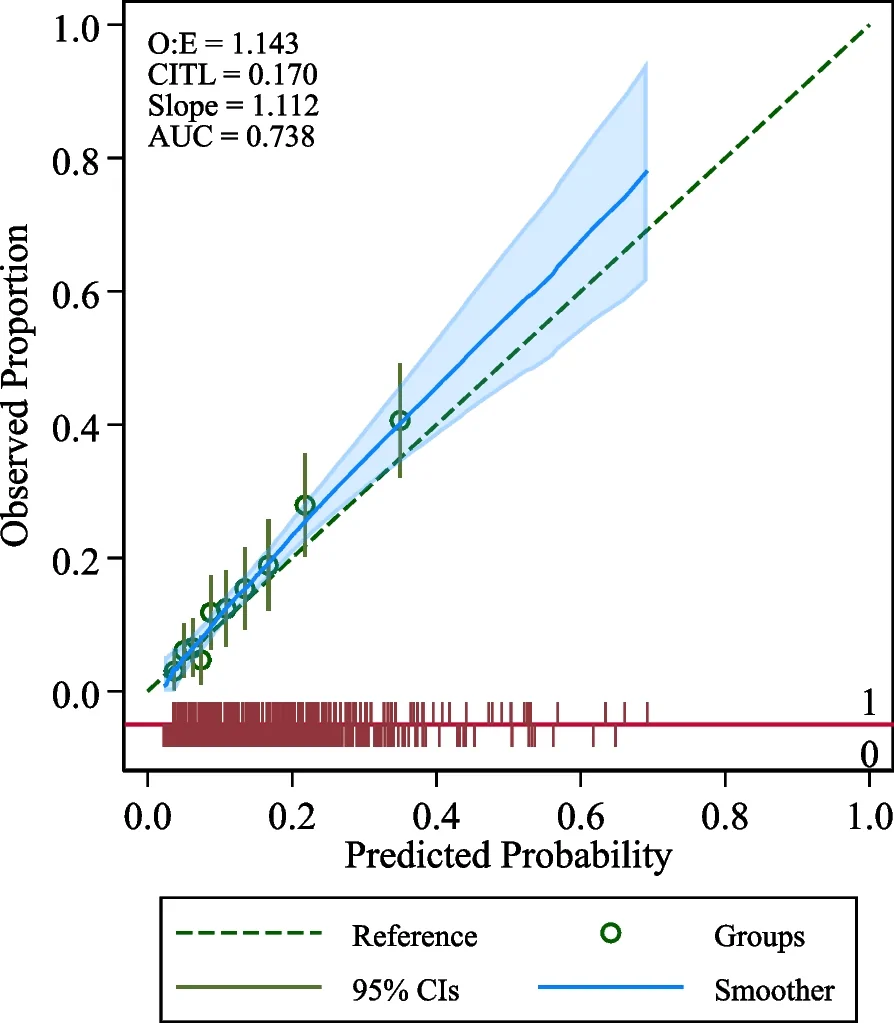
Calibration Plot for the Prediction Model in the Temporal Validation Cohort. Predicted probabilities of suicidal ideation were compared with observed proportions across deciles of risk. The plot demonstrates overall good agreement between predicted and observed risks, with no evidence of systematic over‑ or underestimation

Model calibration was assessed using decile‑based calibration plots comparing predicted probabilities with observed proportions of suicidal ideation. Overall, the predicted risks agreed well with the observed outcomes in the validation cohort. Although some variability was observed in the higher predicted risk range, no systematic over‑ or underestimation of risk was evident (Fig. 3).

### Decision curve analysis

**Fig. 4:**
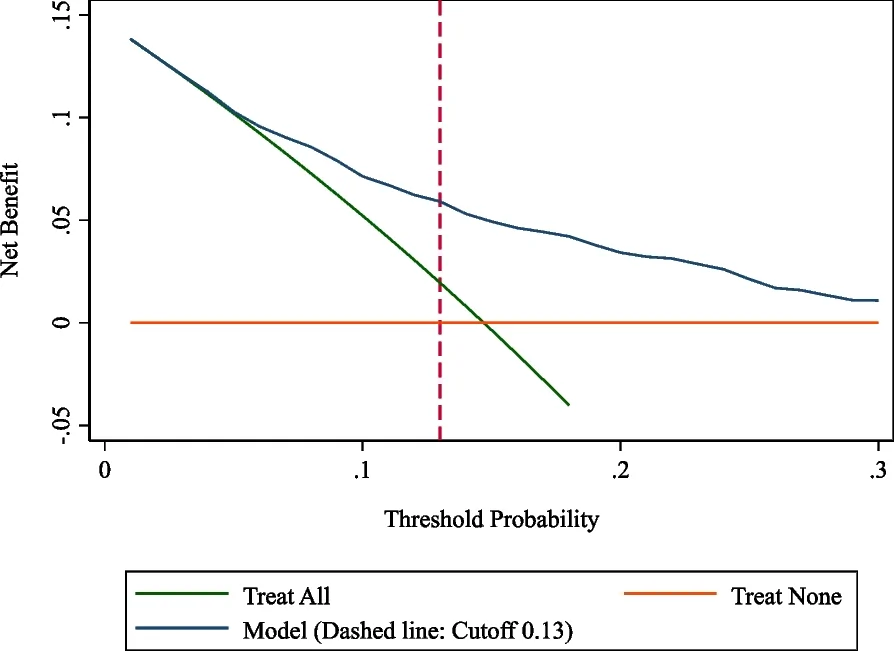
Decision Curve Analysis of Potential Clinical Utility of Prediction Model in the Temporal Validation Cohort. Net benefit is shown across a range of clinically plausible threshold probabilities. Use of the prediction model provided greater net benefit than default strategies of intervening in all students or intervening in none. The vertical line indicates the Youden‑based reference threshold

DCA was performed in the validation cohort to evaluate the potential clinical utility of the prediction model. Across a range of clinically plausible threshold probabilities, the prediction model demonstrated greater net benefit than default strategies of either intervening in all students or in none (Fig. 4).

At a threshold probability corresponding to the Youden-based cutoff (approximately 0.13), use of the prediction model was associated with a positive net benefit, supporting its potential value for risk stratification at the time of first contact with university counseling services.

### Summary of model performance

Overall, the prediction model demonstrated stable discrimination, acceptable calibration, and favorable decision‑analytic performance in the development and temporal validation cohorts. These findings support the robustness of the model and suggest its potential utility for identifying students at increased risk of suicidal ideation at initial counseling intake.

## Discussion

### Principal findings

In this study, we developed and temporally validated a multivariable prediction model for suicidal ideation assessed at the time of first contact with a university counseling service, using information obtained from an annual mental health screening conducted prior to counseling service use. The model demonstrated stable discrimination, acceptable calibration, and favorable decision‑analytic performance across temporally distinct cohorts. These findings indicate that routinely collected screening data can be used to identify students at an elevated risk of suicidal ideation at intake.

A major strength of the model is the clear temporal separation between predictors and outcome. Psychological distress indicators, self‑esteem, lifestyle‑related factors, and demographic characteristics were collected through routine campus‑wide screening independently of counseling service utilization, whereas suicidal ideation was assessed later at intake. This temporal ordering minimizes information leakage and supports interpretation of the model as a screening‑based prediction tool rather than a cross‑sectional diagnostic aid.

Among screening‑based predictors, higher levels of psychological distress—particularly feelings of worthlessness—were consistently associated with increased odds of suicidal ideation at intake. Female sex and lower self‑esteem were also associated with higher odds of suicidal ideation, whereas access to the counseling service via call‑based or early outreach routes was associated with lower odds.

Lifestyle‑related factors, such as lack of regular exercise and skipping breakfast, were not independently associated with suicidal ideation in the multivariable model, but were retained as theoretically relevant covariates to support overall risk adjustment and model stability.

The finding regarding call-based or early outreach access requires further consideration. This is supported by the supplementary analyses of the screening population and service utilization. As shown in Table 2, the overall proportion of students identified as high risk based on screening criteria remained relatively stable across different academic years, whereas patterns of help-seeking behavior varied substantially. In particular, voluntary visits were more common in the pre-pandemic period (2017–2019), whereas call-based outreach accounted for a larger proportion of counseling access in 2021. These findings indicate that the observed protective association of call-based access may not simply reflect lower-risk individuals; rather, it may represent the role of outreach as a mechanism to engage students who might otherwise not seek help. An alternative explanation is that outreach-based contact may facilitate earlier engagement before symptoms escalate, considering that students who seek help voluntarily may do so after experiencing more severe distress, which could result in higher levels of suicidal ideation at intake. These interpretations are consistent with the shift in help-seeking behavior observed during the COVID-19 pandemic, when barriers to voluntary service use may have increased, thereby elevating the relative importance of proactive outreach strategies. This perspective on engagement could also help explain why certain lifestyle-related variables were retained in the model although they lacked statistical significance, given their potential role as accessible entry points for engagement in real-world counseling settings. In practice, such factors may serve as less intrusive and more approachable entry points during outreach-based or early contact interventions, compared with direct interventions addressing sensitive psychological symptoms. Therefore, even in the absence of statistical significance, such variables may facilitate engagement with students and contribute to the practical usefulness of the model without compromising its overall performance.

Taken together, the observations at the individual and system levels suggest that the COVID-19 pandemic had a greater impact on help-seeking behavior and access pathways than on the overall distribution of screening-based psychological risk. The proportion of high-risk individuals remained relatively stable; however, the pathway through which students accessed counseling services shifted, with call-based outreach playing a more prominent role when voluntary visits declined. These findings highlight the importance of considering both individual- and system-level factors when interpreting and applying such models.

### Comparison with previous research

Previous studies examining suicidal ideation among university students have often relied on cross‑sectional data in which psychological symptoms and suicidal thoughts were assessed simultaneously, limiting interpretation of temporal relationships [27–30, 33]. In contrast, the present study leveraged routinely collected screening data obtained before the use of counseling services to predict suicidal ideation assessed later at intake. This design strengthens the evidence that elevated psychological distress and low self‑esteem identified during screening are meaningful indicators of subsequent risk at the point of help‑seeking.

The importance of worthlessness and related cognitive‑affective symptoms observed in this study is consistent with prior research linking negative self‑evaluation and hopeless cognitions to suicidal ideation [30, 32]. However, by embedding these variables within a prediction modeling framework and validating the model temporally, the present study extends existing literature from explanatory associations toward practical risk stratification.

### Clinical and practical implications

From a practical perspective, the findings suggest that annual mental health screening data may be used to support the early identification of students who present to counseling services with elevated risk of suicidal ideation. The Youden Index was used to identify a reference probability threshold that balanced sensitivity and specificity in the development cohort. This threshold was not intended to function as a rigid clinical cutoff but rather to facilitate interpretation of model performance and downstream evaluation of clinical utility.

DCA further demonstrated that, across a range of clinically plausible threshold probabilities—including the Youden‑based reference point—the prediction model yielded greater net benefit than default strategies of intervening in all students or intervening in none. This indicates that using model‑estimated risk could, in principle, improve the efficiency of targeted risk assessment or follow‑up without increasing unnecessary interventions. The model is intended to support, rather than replace, clinical judgment within existing student support systems.

As all predictors and their corresponding coefficients are explicitly specified, the model can be reproduced in other institutions using comparable screening data. However, its applicability may depend on the similarity of screening procedures, variable definitions, and student populations. The model may be applied to identify students at elevated risk of suicidal ideation based on screening data and to support prioritization for outreach or early intervention.

### Strengths

This study has several notable strengths. First, the clear temporal ordering of predictors and outcome enhances interpretability and reduces the likelihood of information leakage, which is a common limitation in mental health prediction studies. Second, the use of routinely collected screening data improves feasibility and potential scalability in real‑world university settings. Third, we conducted temporal external validation and DCA, providing evidence of not only predictive performance but also potential clinical utility. In addition, predictors were selected a priori and entered using a forced‑entry approach, reducing the risk of overfitting associated with data‑driven variable selection. Together, these features support the robustness and practical relevance of the proposed model.

### Limitations

Several limitations should be acknowledged. First, this study was conducted within a single university counseling service, which may limit its generalizability to other institutions or cultural contexts. Second, suicidal ideation was assessed using a single intake item and may not capture the full complexity or persistence of suicidal thoughts over time. Third, to enhance robustness and interpretability, selected psychological distress measures were dichotomized; although exploratory analyses supported this approach, some information loss is inevitable. Finally, although the model demonstrated stable performance across temporal cohorts, additional external validation in independent populations and prospective evaluation are required before routine implementation.

In addition, Table 2 shows that a substantial proportion of students identified as high risk in the screening did not attend counseling services despite outreach efforts. This finding may be attributable to potential selection bias, as the present analyses were restricted to students who completed the screening survey and subsequently accessed counseling services. Therefore, the study findings should be interpreted within this specific context and may not fully represent the entire screened student population.

## Conclusions

In conclusion, this study demonstrates that routinely collected mental health screening data can be used to predict suicidal ideation with reasonable accuracy and potential clinical utility at the time of initial counseling contact. Screening‑based prediction models of this kind may complement existing student support systems by facilitating earlier recognition of elevated risk and informing timely, proportionate intervention strategies.

## Data Availability

The datasets generated and analyzed during the current study are not publicly available due to institutional privacy regulations but are available from the corresponding author on reasonable request.

## Abbreviations

AUC: Area Under the Receiver Operating Characteristic Curve
CI: Confidence Interval
DCA: Decision Curve Analysis
K10: Kessler Psychological Distress Scale (10-item)
OR: Odds Ratio
RSES: Rosenberg Self-Esteem Scale (10-item)
ROC: Receiver Operating Characteristic
SD: Standard Deviation
SE_total: Total score of Self-esteem (Rosenberg Self-Esteem Scale)
VIF: Variance Inflation Factor
